# Umbelliprenin isolated from *Ferula sinkiangensis* inhibits tumor growth and migration through the disturbance of Wnt signaling pathway in gastric cancer

**DOI:** 10.1371/journal.pone.0207169

**Published:** 2019-07-01

**Authors:** Lijing Zhang, Xiaobo Sun, Jianyong Si, Guangzhi Li, Li Cao

**Affiliations:** 1 Institute of Medicinal Plant Development, Chinese Academy of Medical Sciences & Peking Union Medical College, Beijing, China; 2 Institute of Food Science and Technology, Chinese Academy of Agricultural Sciences (CAAS), Beijing, China; Chung Shan Medical University, TAIWAN

## Abstract

The traditional herb medicine *Ferula sinkiangensis* K. M. Shen (*F*. sinkiangensis) has been used to treat stomach disorders in Xinjiang District for centuries. Umbelliprenin is the effective component isolated from *F*. *sinkiangensis* which is particularly found in plants of the family Ferula. We previously reported the promising effects of Umbelliprenin against gastric cancer cells, but its anti-migration effect remained unknown. Here we investigated the anti-migration effect and mechanism of Umbelliprenin in human gastric cancer cells. In SRB assay, Umbelliprenin showed cytotoxic activities in the gastric cancer cell lines AGS and BGC-823 in a dose-and-time-dependent manner, while it showed lower cytotoxic activity in the normal gastric epithelium cell line GES-1. During transwell, scratch and colony assays, the migration of tumor cells was inhibited by Umbelliprenin treatment. In gelatin zymography assay, Umbelliprenin could inhibit the expression of MMP2 and MMP9 in tumor cells The expression levels of the Wnt-associated signaling pathway proteins were analyzed with western blots, and the results showed that Umbelliprenin decreased the expression levels of proteins of the Wnt signalling pathway, such as Wnt-2, β-catenin, GSK-3β, p-GSK-3β, Survivin and c-myc. The translocation of β-catenin to the nucleus was also inhibited by Umbelliprenin treatment. In TCF reporter assay, the transcriptional activity of T-cell factor/lymphoid enhancer factor (TCF/LEF) was decreased after Umbelliprenin treatment. The in *vivo* results suggested that Umbelliprenin induced little to no harm in the lung, heart and kidney. Overall, these data provided evidence that Umbelliprenin may inhibit the growth, invasion and migration of gastric cancer cells by disturbing the Wnt signaling pathway.

## Introduction

Gastric cancer is the fourth most common cancer worldwide and is one of the most prevalent cancers among men in China [1,2], which may be related to their preference for pickled food and red meat [3]. The clinical outcome of gastrointestinal cancer surgery is limited because of the high rates of postoperative complications [4], such as the systemic inflammatory response [5], which may increase tumor recurrence after surgery [6]. Therefore, studies have focused on raising the overall survival rate for gastric cancer [7]. However, only trastuzumab and ramucirumab have been approved for the treatment of advanced gastric cancer as targeted monoclonal antibodies [8], suggesting that less toxic and more effective therapeutic options are necessary.

Metastasis is the main cause of death in cancer patients [9]. The invasion of cancer cells is affected and modulated by many biological molecules and signaling pathways. Growing evidence indicates that there are abnormalities in the Wnt pathway in human gastric cancer [10]. Without the Wnt signals, β-catenin in the cytosol is continuously degraded. Instead, with the Wnt signals, the level of β-catenin increases, β-catenin in the cytosol accumulates and then translocates to the nucleus and, binds to the T-cell factor (TCF)/lymphoid enhancer factor (LEF) [11]. These transcription factors regulate the expression of specific downstream target genes such as c-myc and Survivin, which are involved in oncogenesis [11–13]. Therefore, the components of the Wnt signalling pathway could be good therapy targets for gastric cancer.

Natural products have been used as traditional medicines for gastric cancer therapy [14]. Ferula sinkiangensis K. M. Shen is a traditional folk medicine, which has been used for treating stomach disorders in Xinjiang District since the Tang Dynasty. Umbelliprenin is an effective component of F. sinkiangensis and exhibits anti-cancer effects in many cancer cells lines [15,16]. Our previous studies have first explored the growth inhibition effect of Umbelliprenin in gastric cancer cells: this compound could induce apoptosis and cell cycle arrest [17]. In the present report, we reported that Umbelliprenin can inhibit tumor growth and migration, and that the underlying anticancer mechanism of Umbelliprenin is associated with the Wnt signal pathway.

## Materials and methods

### Reagents and antibodies

Dulbecco’s Modified Eagle’s Medium (DMEM), Ham’s F12 medium, trypsin, penicillin, streptomycin and fetal bovine serum (FBS) were purchased from Gibco (CA, USA). Sulforhodamine B (SRB) and Dimethyl Sulphoxide (DMSO, purity>99.9%) were purchased from Sigma-Aldrich (MO, USA). Transwell systems were purchased from Corning (NY, USA). Matrigel basement membrane matrix was bought from BD Biosciences (NJ, USA). M50 Super 8x TOPFlash (12457), M51 Super 8x FOPFlash (TOPFlash mutant) (12457) and pRL-SV40P (27163) were obtain from Addgene. Lipofectamine 2000 was bought from Thermo (MA, USA). The dual luciferase reporter assay kit was obtained from KeyGEN Biotech (Jiangsu, China). The nuclear and cytoplasmic protein extraction kit was purchased from Beyotime Biotechnology (Jiangsu, China). Antibodies against Wnt-2, GSK-3β, c-myc, β-catenin, p-GSK-3β, Survivin, Lamin B and β-actin were purchased from Cell Signaling Technology (MA, USA). The cECL Western Blot Kit was obtained from CoWin Biotech (Beijing, China). All the chemical reagents were of the highest grade.

### Cell culture and compounds

AGS (human gastric carcinoma cells) cell line was purchased from national infrastructure of cell line resource (China). GES-1 (human normal gastric epithelial cell) cell line was purchased from Cancer Institute & Hospital, Chinese Academy of Medical Sciences (China). BGC-823 (human gastric cancer cells) cell line was from Chinese Academy of Sciences (China). AGS was cultured in Ham’s F12 medium containing 10% FBS, 100 U/mL penicillin, and 100 μg/mL streptomycin at 37°C with 5% CO_2_. GES-1 and BGC-823 were cultured in DMEM supplemented with 10% FBS, 100 U/mL penicillin and 100 μg/mL streptomycin under the same conditions. Cells were passaged at least three times before being used in experiments. Umbelliprenin was obtained from the seeds of *F*. *sinkiangensis* as previously described [17]. Briefly, the seeds of *F*. *sinkiangensis* were collected from Yili state, Xinjiang Uygur Autonomous Region of China. Then Professor Jianyong Si (one of our authors) from Institute of Medicinal Plant Development isolated the compounds Umbelliprenin from the seed. The compound was identified by spectra methods (UV, IR, MS and NMR) and purified by HPLC (purity >90%). And the molecular formula of umbelliprenin is C_24_H_30_O_3_. Then we used dimethyl sulfoxide to dissolve Umbelliprenin as stock solutions and dilute with medium prior to use to ensure the final concentration of DMSO was less than 0.1% (v/v).

### Animals

Six-week-old male BALB/c nude mice were obtained from the Vital River Laboratories (Beijing, China) and maintained in a 12 h light/dark cycle environment (25 ± 2°C) where they received and food ad libitum. The protocol for the animal experiments was approved by the Animal Ethics Committee at the Institute of Medicinal Plant Development, Chinese Academy of Medical Sciences.

### Cell viability assay

The Sulforhodamine B (SRB) assay was used to determine cell viability. Cells were seeded in 96-well plates in triplicate and cultured for 24 h at 37°C. Then, the cells were treated with Umbelliprenin in various concentrations (0, 3.125, 6.25, 12.5, 25, 50 μM). After the 24 h treatment, 50 μL of cold TCA was added to fix the cells for 1 h at 4°C. The plates were washed with water and air-dried. One-hundred microlitres of 0.4% (w/v) SRB was added and cells were stained for 15 min. After washing 4 times with acetic acid, 100 μL of Tris-base was added for 10 min, while shaking. The absorbance was measured at 540 nm using a Microplate Reader (Bio Tek, USA). % Cytotoxicity = (Control–Experimental) / Control*100%.

### Colony formation assay

The colony formation assay was used to evaluate the anchorage-independent growth of gastric cancer cells. Cells were seeded in 6-well plates and treated with Umbelliprenin (0, 6, 12, 24 μM for AGS cells and 0, 12.5, 25, 50 μM for BGC-823 cells) for 10 days. The tumor colonies were observed counted using a microscope. Then, the colonies were stained with crystal violet (1 mg/mL), and colonies larger than 200 μm were counted.

### Wound healing assay

Cell motility was detected using the wound healing assay. Cells were cultured in a 24-well plate to nearly 90% confluence. The monolayers were then carefully scratched using a sterile 200-μL pipette tip with a constant width. The cells were washed with PBS and treated with Umbelliprenin (IC50 values were used in both AGS and BGC-823 cells). The cells were photographed at 0, 24, 36 and 48 h after treatment to observe the distances that the cells had migrated. The cell motility was calculated as follows: Cell motility = (distance at 0 h—distance at 24, 36 or 48 h) / distance at 0 h*100%.

### Transwell-migration / invasion assays

Cell migration was analyzed in a 24-well transwell plate with 8-μm pore size polyvinylidene filter membrane. Cells were seeded into the upper chamber (1×105) in serum-free Ham’s F12 medium and treated with Umbelliprenin (0, 6, 12, 24 μM for AGS cells and 0, 12.5, 25, 50 μM for BGC-823 cells). Ham’s F12 medium with 10% serum was added to the lower chamber. After incubation for 24 h, the cells on the upper side of the filter were removed with cotton swabs, and the filters were fixed with 4% formaldehyde for 10 min at room temperature, and stained with crystal violet for 15 min, the number of cells in five random fields of each triplicate filter was counted under the light microscope. The cell invasion assay was conducted under similar procedure, except that 80 μL of Matrigel was used to coat the upper chamber for 12 h before the cells were seeded.

### Matrix metalloproteinases assay

The enzymatic activity of the matrix metalloproteinases MMP2 and MMP9 was examined by gelatin zymography. Briefly, cells were treated with Umbelliprenin (0, 6, 12 and 24 μM for AGS cells or 0, 12.5, 25 and 50 μM for BGC-823 cells) for 24 h and the medium was then collected and centrifuged 3500 rpm for 10 min. The proteins in the samples were separated by SDS-PAGE. After electrophoresis, the gel was washed twice with 50 mM Tris-HCl buffer with 2.5% Triton X-100 (pH 7.6) and then incubated with a reaction buffer (20 mM Tris-HCl, 5 mM CaCl2, 1 mM ZnCl2, pH 7.6) at 37°C for 36 h. The results were subsequently obtained after staining and washing.

### β-Catenin / TCF transcription reporter assay

The TCF-reporter plasmids (TOPFLASH and the negative control FOPFLASH) and Renilla luciferase (pRL-SV40P) plasmid were obtained from Addgene. The reporter plasmids containing TCF binding sites (TOPFLASH, 500 ng/well) or inactive TCF binding sites (FOPFLASH, 500 ng/well) were transfected into the cells for 6 hours using Lipofectamine 2000. The cells were co-transfected with the Renilla luciferase (pRL-SV40P, 5 ng/well) plasmid to normalize the transfection efficiency with the internal control. Then, the medium was renewed and different concentrations of Umbelliprenin were added. After the 24 h treatment, TCF-mediated gene transcription was expressed by the ratio of TOPFLASH: FOPFLASH luciferase activity, and each value was normalized to the Renilla luciferase activity.

### Protein isolation and Western blot

Fractionated nuclear and cytosolic proteins were obtained according to the manufacturer’s instructions. Briefly, cells were treated with Umbelliprenin (0, 6, 12, 24 μM for AGS cells and 0, 12.5, 25, 50 μM for BGC-823 cells) for 24 h, harvested and washed twice with ice-cold PBS. 500 μL of cytoplasmic extract agent A and B were added and the suspension was centrifuged at 12,000 rpm for 5 min. The supernatant contained cytosolic proteins. The nuclear residue was mixed with 200 μL of the nuclear extract agent. The mixture was vortexed for 30 min and then centrifuged at 14,000 rpm for 10 min at 4°C. The supernatant contained nuclear proteins.

To gain whole-cell lysates, cells were lysed in lysis buffer after treatment with Umbelliprenin (0, 6, 12, 24 μM for AGS cells and 0, 12.5, 25, 50 μM for BGC-823 cells) for 24 h and the protein concentrations were determined by the BCA method. Protein samples were separated by SDS-PAGE and transferred onto PVDF membranes. After blocking for 1 h in the 5% non-fat milk solution, the membranes were incubated with the primary antibody overnight at 4°C. Then the primary antibody was washed with TBST for 3 times and incubated with secondary antibody for 1 h at room temperature. Protein bands were detected using ECL and the levels of β-actin were used to ensure equal loading of proteins.

### Immunofluorescence staining to detect the nuclear translocation of β-catenin

Cells were cultured in plates and treated with Umbelliprenin (0, 6, 12, 24 μM for AGS cells and 0, 12.5, 25, 50 μM for BGC-823 cells) for 24 h. The treated cells were fixed with 4% paraformaldehyde for 10 min and washed twice with PBS. These cells were blocked with 5% bovine serum albumin in PBS for 60 min, and then incubated with the primary antibody (diluted 1:200 in PBS containing 3% BSA) overnight at 4°C. After washing twice with PBS, the cells were co-incubated with the IgG PE-conjugated secondary antibody (diluted 1:400 in PBS containing 3% BSA) and DAPI for 1 h at room temperature. The cells were examined with the Image Xpress system (Molecular Devices, USA).

### Safety of Umbelliprenin in tumor xenograft models

Animal experiments were conducted according to the previous description [Zhang et al., 2015]. Briefly, mice were inoculated subcutaneously with the human gastric cancer cells BGC-823 (1.0 × 106) on the right flank. The mice were randomized to six groups and their conditions were observed every day. Umbelliprenin were diluted in 200 μL 0.9% NaCl solution and administered to each mouse at a dose of 10 mg/kg or 20 mg/kg twice daily for 12 days. At the end of the experiment, all mice were fasted overnight and then euthanized by CO2 inhalation. and the lungs, livers, hearts and kidneys were fixed and preserved in formalin for hematoxylin-eosin staining. Blood samples were collected and centrifuged at 12,000 rpm for 15 min to obtain the serum. Liver function was evaluated based on the serum levels of aspartate aminotransferase (AST) and alanine aminotransferase (ALT). All biochemical parameters were evaluated by an automated biochemical analyzer (Beckman).

### Statistical analysis

All data were analyzed by **the** IBM SPSS statistics 19 **software**. All tests were conducted at least three times. Statistical significance was defined as *p < 0.05 and **p < 0.01. The results were expressed as mean ± SD which represent three independent tests.

## Results and discussion

### Results

#### Umbelliprenin reduced the viability in AGS and BGC-823 human gastric cancer cells, but not in GES-1 cells

**Fig 1:**
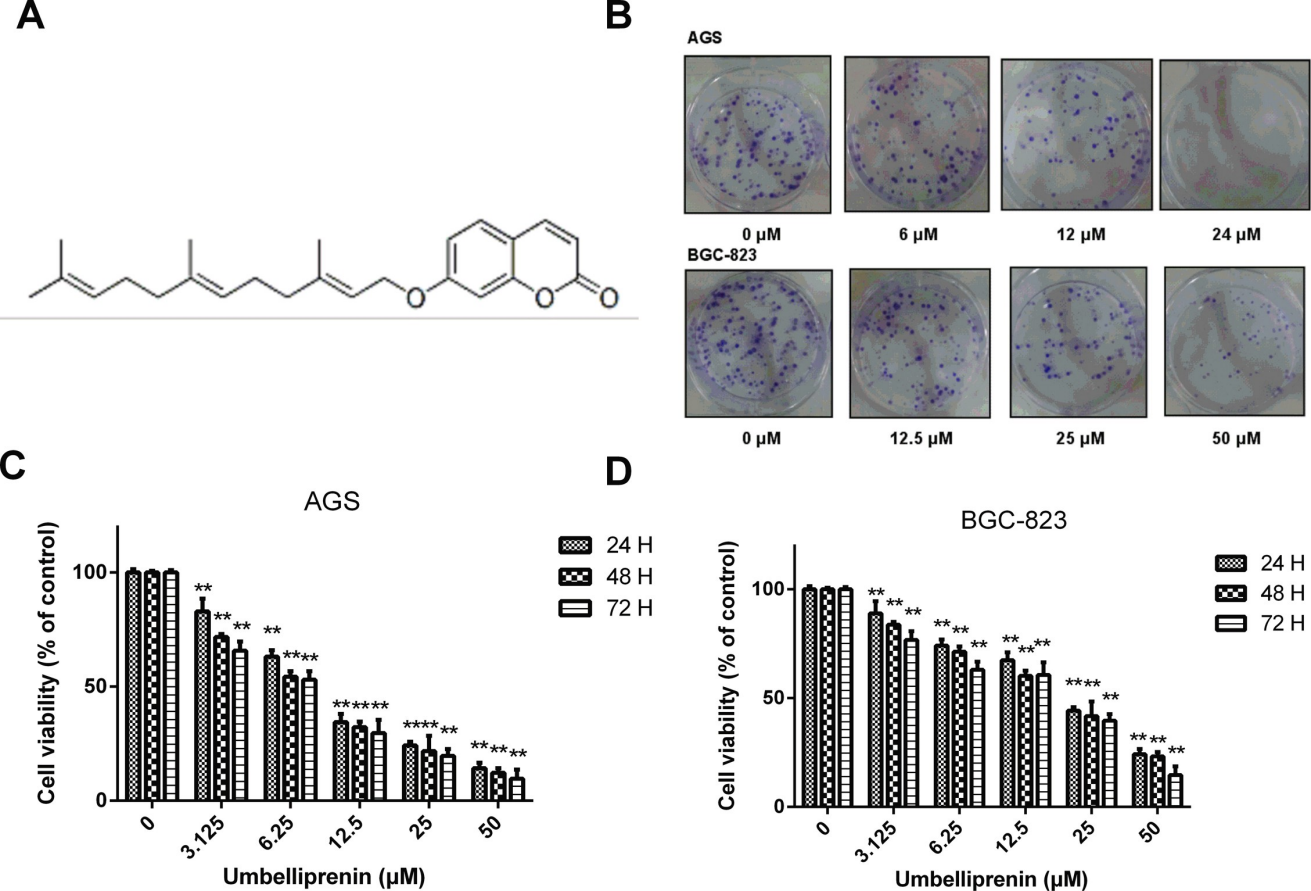
Chemical structure and inhibition effects on cell viability of Umbelliprenin. A. Chemical structure of Umbelliprenin isolated from the seeds of *Ferula sinkiangensis*. B. Colony formation assay. AGS or BGC-823 cells were grown for 10 days after incubation with different concentrations of Umbelliprenin. C. The effects of Umbelliprenin on the viability of AGS human gastric cancer cells. D. The effects of Umbelliprenin on the viabilities of human gastric cancer cells BGC-823. AGS and BGC-823 cells were exposed to various concentrations of Umbelliprenin (0, 3.125, 6.25, 12.5, 25, 50 μM) for 24 h, 48 h or 72 h, followed by the Sulforhodamine B (SRB) assay. The data represent the mean value of three independent experiments and are expressed as the mean ± SD. **p < 0.01, *p < 0.05 were considered statistically significant.

The chemical structure of Umbelliprenin isolated from the seeds of F. sinkiangensis is shown in Fig 1A. Because Umbelliprenin appeared to be most effective in gastric cancer cells among other common cancer cells, we studied its anti-proliferative effects in the two human gastric cancer cell lines: AGS and BGC-823 as well as in the human gastric epithelial cell line GES-1. The results showed that Umbelliprenin significantly inhibited the colony forming ability of AGS cells in a dose-dependent manner, while it was less effective in BGC-823 cells (Fig 1B). The IC50 values for the three cancer cell lines varied. The results showed that Umbelliprenin inhibited the growth in both the AGS and BGC-823 gastric cancer cell lines with an IC50 of 11.74 μM and 24.62 μM, respectively, the IC50 for AGS was lower than that for the BGC-823 cells (Fig 1C and 1D). In addition, Umbelliprenin was less cytotoxic in GES-1 cells (IC50: 97.55 μM) compared with AGS and BGC-823 cells (Table 1). Based on the IC50 values, we chose 12 μM and 24 μM Umbelliprenin, as well as their multiples or fractions, as the concentrations for treating AGS and BGC-823 cells, respectively. Furthermore, as the colony formation ability is closely related to tumorigenesis in *vivo*, we detected the impact of Umbelliprenin on the growth of gastric cancer cells. Together, these data indicate that Umbelliprenin may decrease the proliferation and tumor forming ability in AGS and BGC-823 gastric cancer cells.

**Table 1:** Cytotoxicity of Umbelliprenin isolated from the seeds of *Ferula sinkiangensis* in three cell lines.

Compounds	IC50(μM)^a^		
	AGS	BGC-823	GES-1
Umbelliprenin	11.74±1.33	24.62±2.45	97.55±3.81

^a^ IC50 is the concentration of compound causing 50% growth inhibition for each cell line after 24 h treatments. The results represent the mean values of three independent tests.

#### The effects of Umbelliprenin on the invasion and migration in AGS and BGC-823 cells

**Fig 2:**
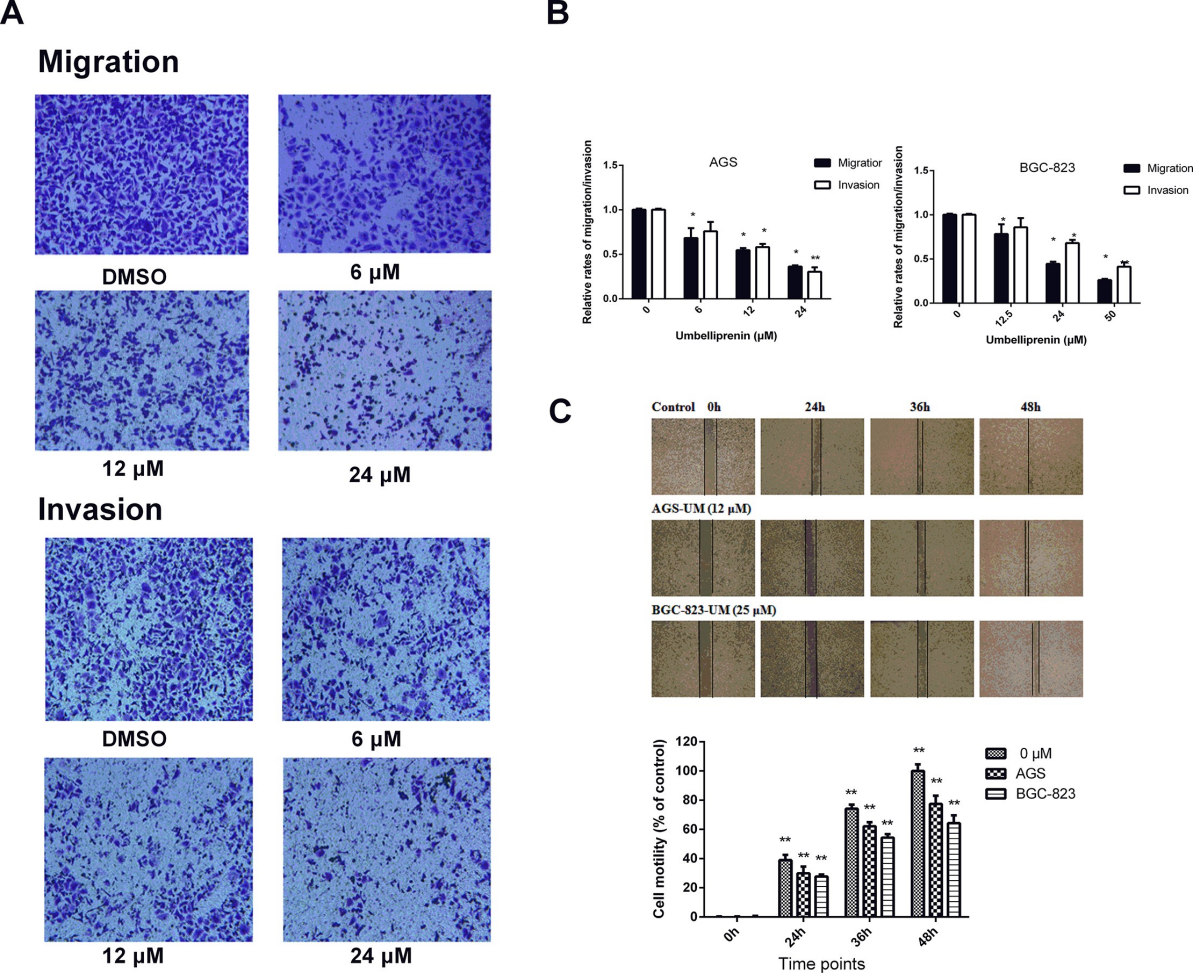
Umbelliprenin inhibits the migration and invasion of gastric cancer cell lines. A. The migration or invasion of cells was measured by transwell or matrigel-coated filter transwell assays, respectively. Cells were incubated with Umbelliprenin for 24 h, and the migrated or invaded cells were fixed and stained with crystal violet. B. The migration and invasion assays data are expressed as the means ± SD and represent the mean value of three independent experiments. **p < 0.01, *p < 0.05. C. The migration of AGS and BGC-823 cells was examined in a wound healing assay. Cells were scratched and treated with Umbelliprenin. Images were obtained using a microscope at 0 h, 24 h, 36 h and 48 h. Data are presented as the mean ± SD and represent three independent experiments. **p < 0.01, *p < 0.05 were considered statistically significance.

Umbelliprenin was effective in reducing cellular migration in gastric cancer cells. The migration assay using the transwell-migration system also showed that Umbelliprenin effectively inhibited the migration of AGS and BGC-823 cells (Fig 2A and 2B). Additionally, Umbelliprenin decreased the invasive potential of cells in a dose-dependent manner (Fig 2A and 2B). Furthermore, the inhibitory effect of Umbelliprenin on the invasion of gastric cancer cells was examined in a transwell assay using Matrigel-coated filters. Compared with the control group, fewer AGS and BGC-823 cells penetrated the filters after Umbelliprenin treatment. Therefore, both the migration and invasion of gastric cancer cells were significantly suppressed after Umbelliprenin treatment. We performed a wound healing assay, and images representing the migration capability of the cells were taken at different time points, at the same site and magnification. Forty-eight hours after scratching, in the presence of Umbelliprenin at its IC50 value, the wound was healed approximately by 77.4% and 64.2% for AGS and BGC-823 cells compared with the control group, respectively (Fig 2C).

#### Umbelliprenin inhibits the expression of MMP2 and MMP9 in AGS and BGC-823 cells

**Fig 3:**
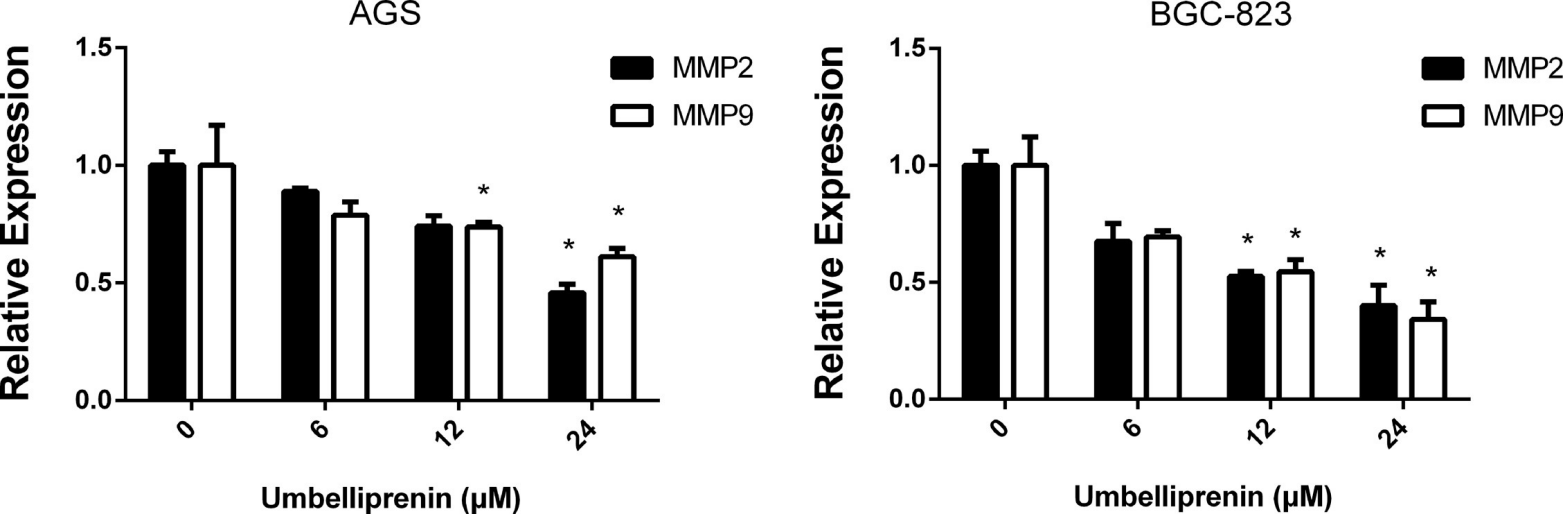
Umbelliprenin treatment decreased the expression of MMP2 and MMP9 in gastric cancer cells. The protein levels of MMP9 and MMP2 were analyzed by gelatin zymography in AGS and BGC-823 cells after Umbelliprenin treatment (0, 6, 12 and 24 μM for AGS cells or 0, 12.5, 25 and 50 μM for BGC-823 cells). The data represent the mean of three individual experiments. *P < 0.05 and **P < 0.01 were considered statistically significant.

As shown in Fig 3, the protein expression levels of MMP2 and MMP9 were investigated in a gelatin zymography assay after Umbelliprenin treatment. Data showed that the expression of MMP2 and MMP9 in the two gastric cancer cells significantly declined after treatment, compared with the control group. These results suggest that Umbelliprenin can regulate the expression of MMP9 and MMP2 in both AGS and BGC-823 cells.

#### Umbelliprenin inhibited the transcriptional activity of β-catenin/TCF in the human gastric cancer cell lines AGS and BGC-823

**Fig 4:**
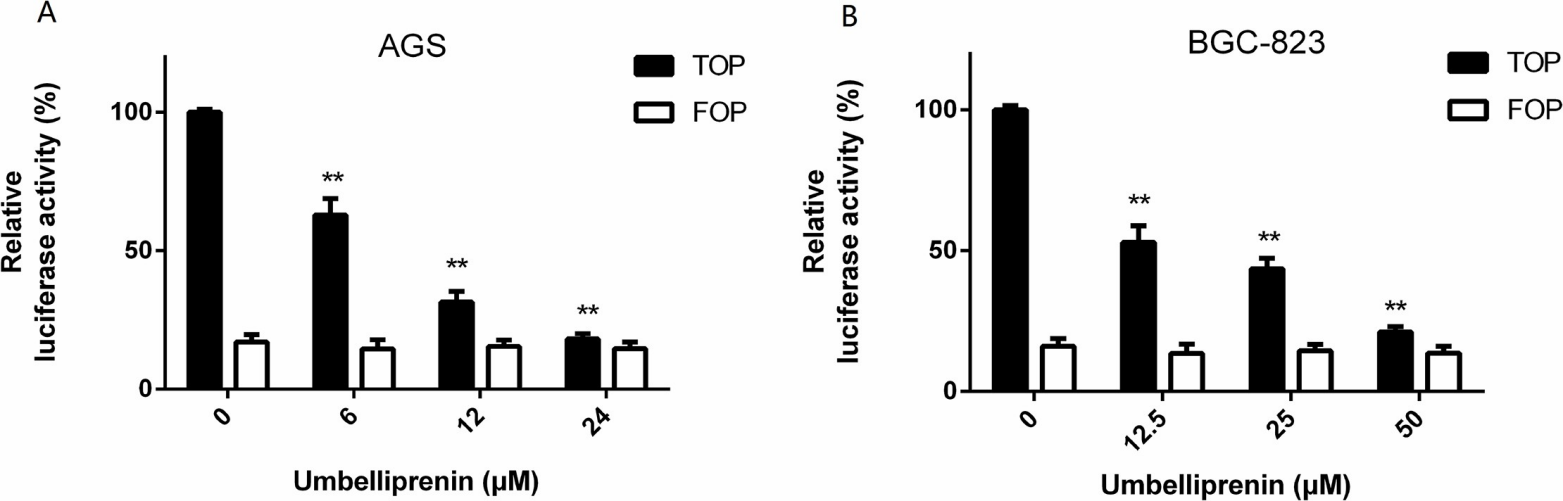
Umbelliprenin treatment decreases the activity of the TCF reporter in gastric cancer cells. A. and B. AGS and BGC-823 cells were transfected with the TOPFLASH or FOPFLASH plasmid together with the Renilla plasmid as control. Cells were then treated with Umbelliprenin (0, 6, 12, 24 μM for AGS cells or 0, 12.5, 25, 50 μM for BGC-823 cells) for 24 hours. The luciferase activity was measured and the results were normalized. The data represent the mean value of three individual experiments. *p < 0.05 and **p < 0.01 were considered statistically significant.

To explore whether Umbelliprenin plays a role on the dysregulation of the Wnt/β-catenin pathway in gastric cancer cells, we examined the transcriptional activity of β-catenin/TCF after Umbelliprenin treatment. For that purpose, we transfected the TOPFLASH or FOPFLASH plasmids into AGS and BGC-823 gastric cancer cells. The transfection efficiency was normalized with Renilla luciferase. As shown in Fig 4A and 4B, after 24 h of treatment Umbelliprenin reduced the TCF-dependent luciferase activity (TOPflash) in a dose-dependent manner in both gastric cancer cell lines, while it did not change the activity of the FOPflash control plasmid. In AGS cells, the transcriptional activity of TCF was 62.93%, 31.54%, and 18.19% relative to the control group at concentrations of 6, 12 and 24 μM of Umbelliprenin, respectively (P<0.05). In BGC-823 cells, the transcriptional activity of TCF was 52.91%, 43.50%, and 21.15% relative to the control group at concentrations of 12.5, 25 and 50 μM of Umbelliprenin, respectively (P<0.05). This indicates that the transcriptional activity of β-catenin/Tcf could be inhibited by Umbelliprenin. Thus, Umbelliprenin suppresses the Wnt/β-catenin signaling pathway in the two gastric cancer cell lines.

#### Umbelliprenin inhibited the nuclear translocation of β-catenin

**Fig 5:**
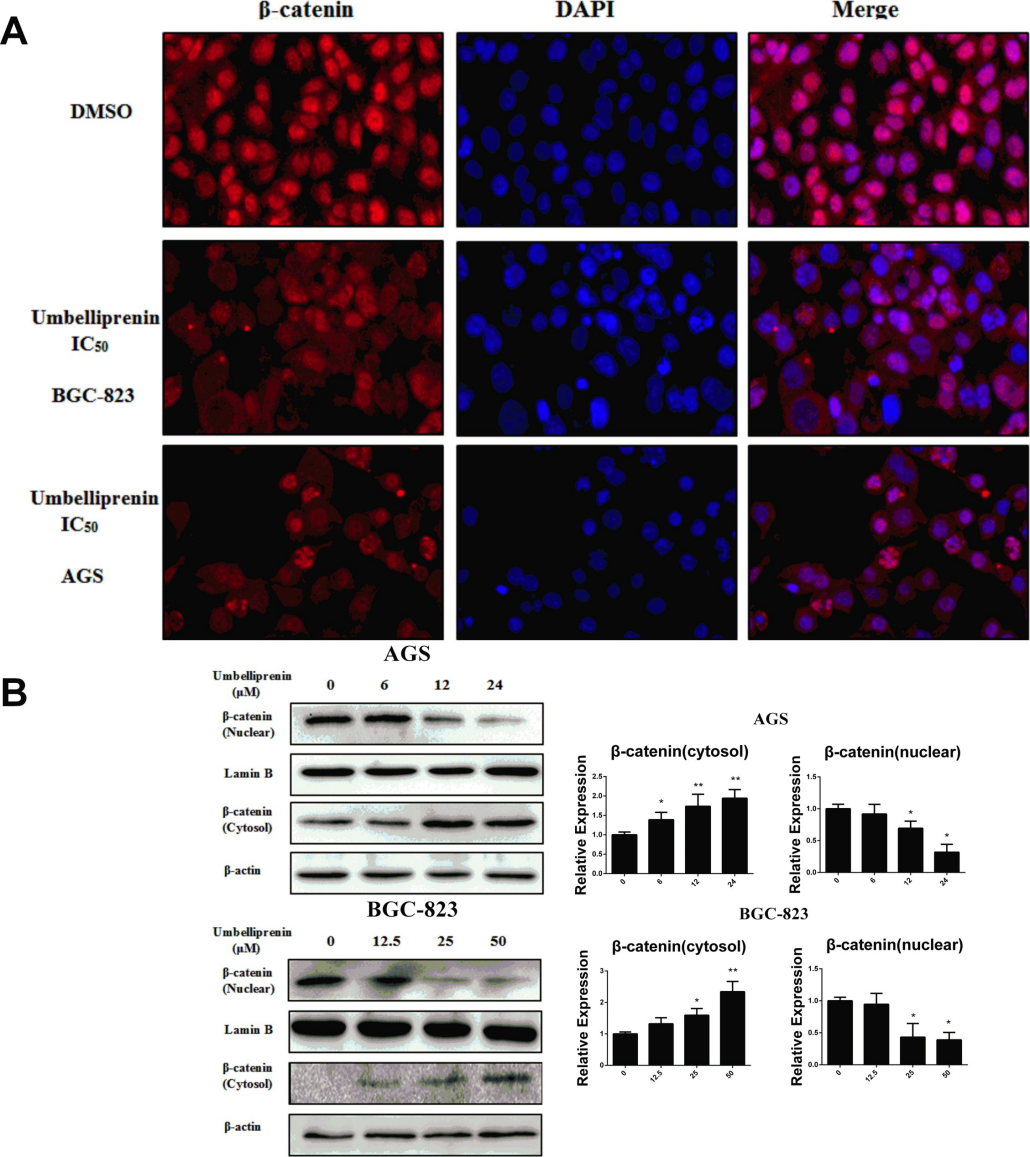
Umbelliprenin inhibits the nuclear translocation of β-catenin in gastric cancer cells. A. The translocation of β-catenin was examined by immunostaining. Cells were treated with UM (0, 6, 12, 24 μM for AGS cells or 0, 12.5, 25, 50 μM for BGC-823 cells) for 24 h, stained with the anti-β-catenin antibody and analysed using the Image Xpress Micro imaging system (MD, USA). B. Protein expressions were detected by western blot. After treatment with Umbelliprenin (0, 6, 12, 24 μM for AGS cells or 0, 12.5, 25, 50 μM for BGC-823 cells) for 24 h. Cellular fractionation was carried out to determine the cellular localization of β-catenin. Lamin B and β-actin were used as controls for nuclear fraction and cytoplasmic fraction, respectively. C. The relative expression of cytoplasmic and nuclear proteins was analyzed. The results obtained from a representative experiment are shown (n = 3). Statistical significance was **p < 0.01.

To determine whether Umbelliprenin suppressed β-catenin mediated transcription by interfering with the nuclear translocation of β-catenin, we investigated the β-catenin subcellular localization in Umbelliprenin -treated AGS and BGC-823 cells by immunofluorescence staining. As shown in Fig 5A, β-catenin preferentially accumulated in the nucleus in the control group. By contrast, after treatment with Umbelliprenin for 24 h, the localization of β-catenin decreased in the nucleus but increased in the cytoplasm and at the plasma membrane. This result was confirmed by Western blot analysis in which β-catenin was increased in the cytoplasmic protein fraction and decreased in the nuclear protein fraction of the treated groups (Fig 5B). The relative expressions of cytoplasmic and nuclear proteins were shown in Fig 5B. The uncropped images of the western blot gel were shown in S1 Fig. Therefore, Umbelliprenin may mediate its effect by inhibiting the translocation of β-catenin to the nucleus.

#### Umbelliprenin inhibited Wnt signaling by decreasing the phosphorylation of GSK-3β and reducing the downstream effectors of Wnt signaling

**Fig 6:**
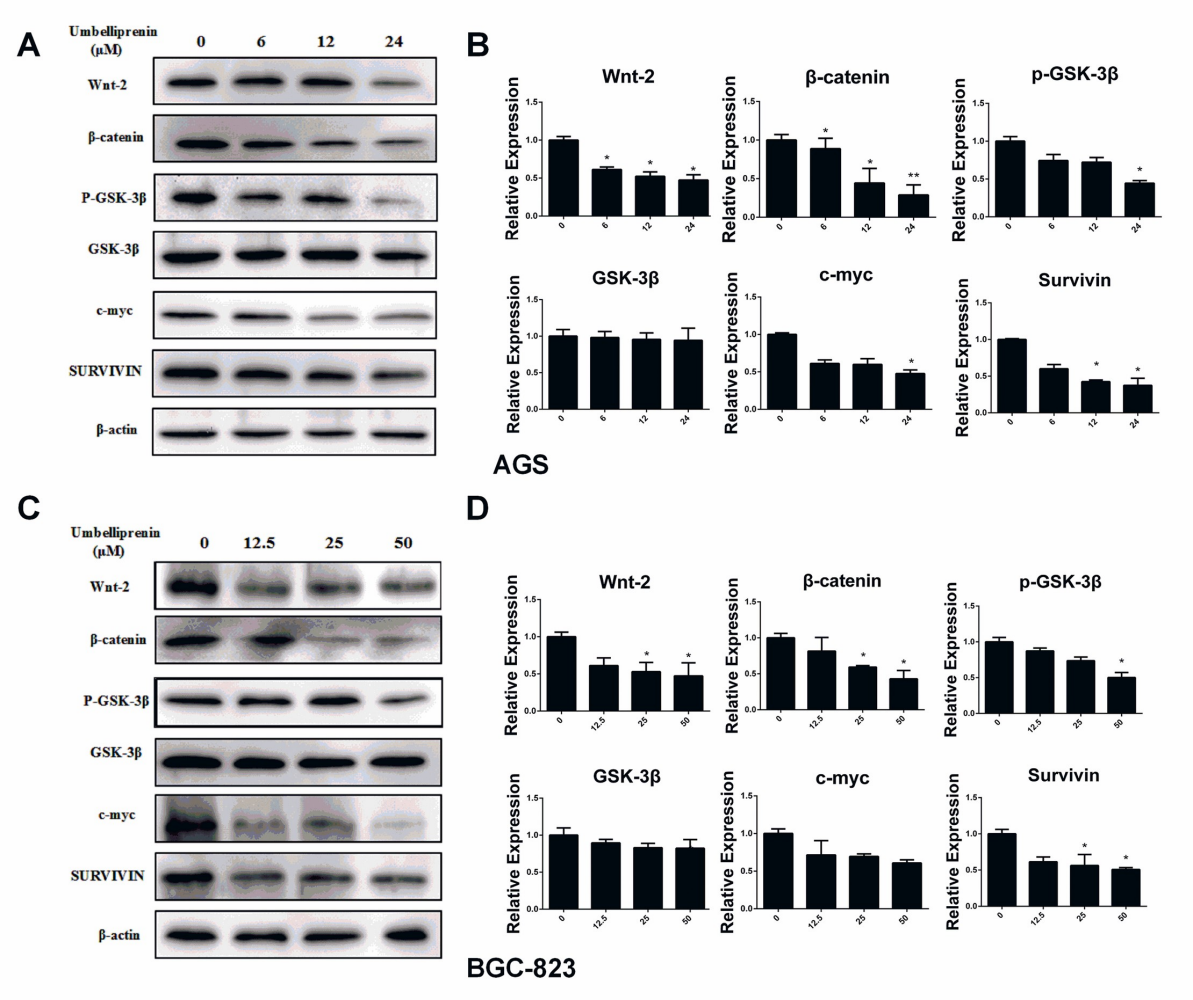
Umbelliprenin downregulates the Wnt signal pathway, and Survivin and c-myc protein expression levels. The expression of the regulatory proteins of the Wnt signal pathway, Survivin and c-myc protein were determined by western blot. A. and C. AGS or BGC-823 cells were treated with Umbelliprenin (0, 6, 12, 24 μM for AGS cells or 0, 12.5, 25, 50 μM for BGC-823 cells) for 24 h. β-actin was used to confirm equal protein loading. B. and D. The relative expression levels of proteins are shown. All tests were performed in triplicate. *p < 0.05 and ** p<0.01 were considered statistically significant.

Wnt signaling substantially impacts gastric tumorigenesis and prognosis [6]. To further determine the mechanisms by which Umbelliprenin inhibits cellular proliferation, migration and invasion, we studied the Wnt signaling. The expression levels of Wnt signaling-associated proteins in both AGS and BGC-823 cells were measured by western blot. Wnt-2, **β**-catenin, and GSK-3**β**, potential modulators of Wnt signaling, as well as the downstream targets of Wnt signaling Survivin and c-myc, were significantly reduced in Umbelliprenin treated cells compared to the control group (Fig 6A and 6C). The relative expressions were shown in Fig 6B and 6D. The uncropped images of the western blot gel were shown in S1 Fig.

#### The safety of Umbelliprenin in BGC-823 tumor xenograft models

**Fig 7:**
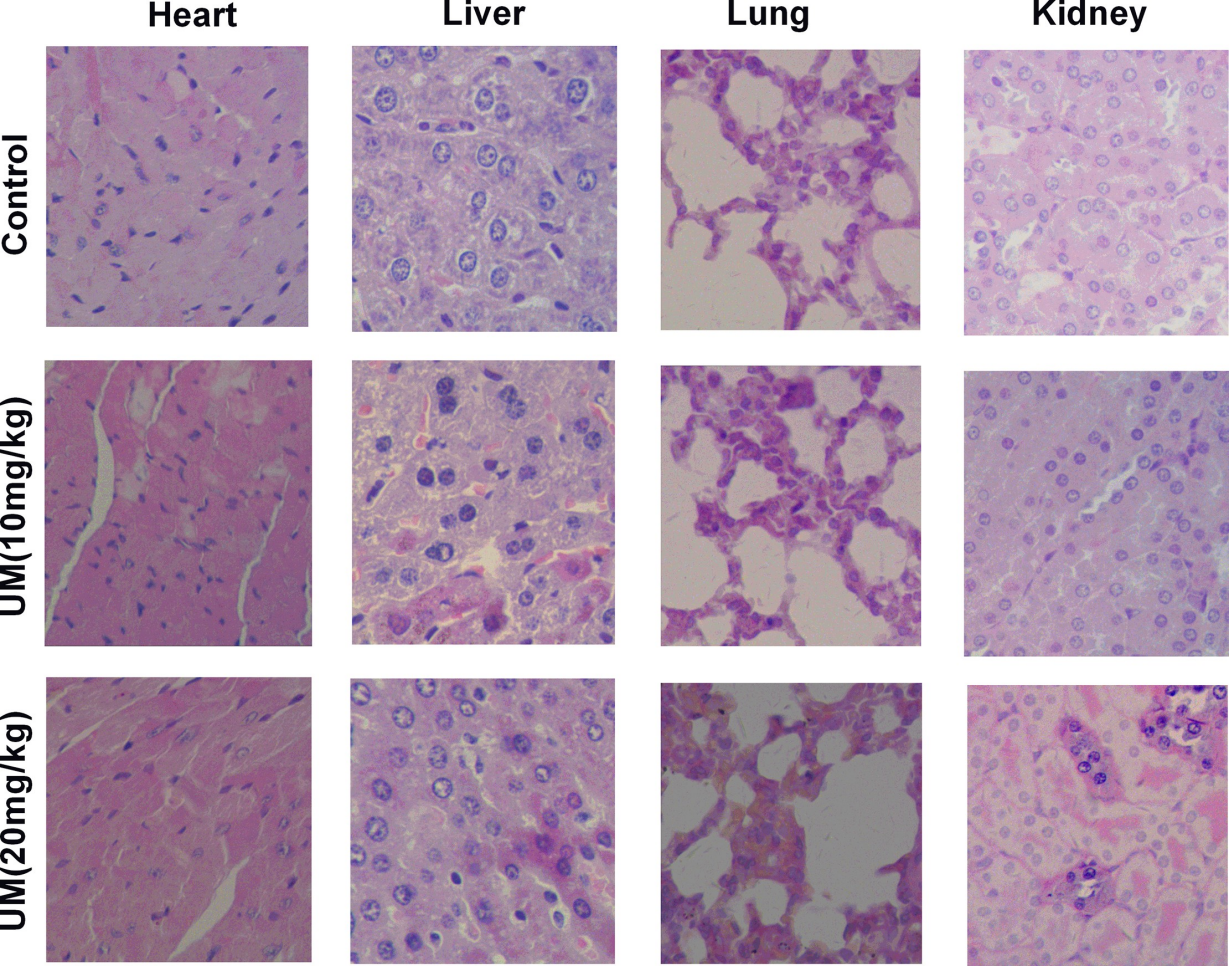
Low toxicity of Umbelliprenin *in vivo*. The results showed representative hematoxylin-eosin staining to investigate the potential toxicity of Umbelliprenin in lung, heart, kidney and liver. The mice were inoculated subcutaneously with the human gastric cancer cells BGC-823 (1.0 × 106) on the right flank. Control group: the mice were administered 0.9% NaCl solution by gavage twice daily at the same volume as Umbelliprenin groups for 12 days; 10 mg/kg group and 20 mg/kg group: the mice were administered 10 or 20 mg/kg Umbelliprenin by gavage twice daily for 12 days. Umbelliprenin were diluted in 200 μL 0.9% NaCl solution. Mice of Umbelliprenin-treated and control mice were then euthanized and hematoxylin-eosin staining were conducted on day 12 (n = 8).

Previous studies showed that Umbelliprenin could effectively inhibit the growth of tumor *in vivo*. Thus, we evaluated the safety of Umbelliprenin in BGC-823 tumor xenograft models. All mice tolerated the treatment procedure well. The analysis of biochemical markers for the liver, as ALT and AST, showed no significant change between the different groups (Table 2). In addition, no histological abnormality was shown in the lungs, liver, heart, and kidneys of mice between the Umbelliprenin treatment groups and control group at the end of the treatment (Fig 7). Together, these data suggest that Umbelliprenin effectively inhibits tumor growth, and does not cause obvious drug-induced adverse effects.

**Table 2:** Serum analysis for liver function (mean ± SD, n = 8).

Group	AST(U/L)	ALT(U/L)
Control	202.6±27.9	31.8±2.3
Umbelliprenin (10mg/kg)	190.4±33.8	30.3±4.1
Umbelliprenin (20mg/kg)	172.9±17.1*	32.1±3.1

*p < 0.05 was considered statistically significance compared with control group. And the biochemical markers ALT and AST were shown no significance between different groups.

## Discussion

Natural products with potential for gastric cancer treatment have received much attention. Umbelliprenin has been reported to possess the promising effect of inducing apoptosis in a variety of cancer cell types such as leukemia, breast cancer and bladder carcinoma [16,18,19] and exhibits biological effects in multiple signal transduction pathways. For example, a study showed that Umbelliprenin could activate both the intrinsic and extrinsic pathways of apoptosis in the Jurkat T-CLL cell line, which accounted for the inhibition of cellular proliferation and apoptosis induction [18]. Therefore, further studies on the anti-gastric cancer effects of Umbelliprenin are necessary. Our previous results showed that Umbelliprenin among seven compounds isolated from the seeds of F. sinkiangensis, exhibits the strongest antitumor effect in vivo and in vitro. Based on these results, we performed further research on Umbelliprenin. In the present study, we demonstrated the anti-metastatic and anti-proliferative effects of Umbelliprenin on the AGS and BGC-823 gastric cancer cell lines in vitro and its safety in vivo. These results indicated that Umbelliprenin might be an effective inhibitor of tumor migration, and were obtained using wound healing, transwell migration and invasion assays. We also demonstrated that the mechanism of action of Umbelliprenin include the inhibition of the Wnt signaling pathways and the decrease of c-myc and Survivin.

The activation of the Wnt/β-catenin signaling is found approximately 30% to 50% of gastric cancer tissues and in many gastric cancer cell lines [20]. β-catenin is a multifunctional protein that was found as an E-cadherin-binding protein involved in the regulation of cell-to-cell adhesion and works as a transcriptional regulator in the Wnt signaling pathway [21]. It accumulates in the cytoplasm and translocates to the nucleus, where it behaves as transcriptional coregulatory factor by interacting with the TCF/LEF complex and activates target oncogenes, such as c-myc and Survivin [22,23]. Our results suggest that Umbelliprenin down-regulates Wnt, resulting in the decrease of phosphorylated GSK-3β and reduction of Wnt downstream effectors, such as β-catenin, Survivin, c-myc, MMP2 and MMP9. Furthermore, Umbelliprenin treatment caused the inhibition of the nuclear translocation of β-catenin and the reduction of the activity of the TCF-reporter. Although results suggest that Umbelliprenin interferes with the Wnt/beta-catenin signaling and therefore inhibits the activity of the TCF reporter, reduction of the reporter activity might be partially caused the Umbelliprenin-induced migration and invasion. Our results are consistent with those previous studies showing that decreased Wnt expression is associated with decreased phosphorylation of GSK-3β; decreased expression of β-catenin, c-myc and Survivin; lower activity of the TCF reporter; and reduced migration and invasion [24].

Taken together, for the first time, we showed strong evidence that Umbelliprenin isolated from the seeds of F. sinkiangensis can inhibit cell growth, migration and invasion, at least in part, through the Wnt signaling pathway. Other reports have shown that the Wnt antagonists inhibit the growth of HCC cells in vitro and in vivo through inhibition of the formation of the TCF-4/β-catenin complex and its transcriptional activity and downregulation of the β-catenin/TCF-4 target genes c-myc and Survivin [25]. By contrast, we found that Umbelliprenin inhibits the Wnt pathway by decreasing nuclear translocation of β-catenin rather than disrupting the TCF-4/β-catenin complex. Therefore, treatments that combine Umbelliprenin with an antagonist of TCF-4/β-catenin may lead to promising effects on suppressing the activation of the Wnt signaling pathway.

## Conclusions

Our data indicate that Umbelliprenin has anti-migration effects in AGS and BGC-823 gastric cancer cells, targets the Wnt signaling pathway, and exhibits good safety during the treatment in the BGC-823 xenograft in *vivo* model: indeed, no abnormalities in regard to body weight, daily diet, liver function and histological characteristics of lung, spleen, heart, kidney and liver tissue were observed. Therefore, Umbelliprenin could be a promising approach for gastric cancer treatment; however, further investigations are necessary.

## Supporting information

S1 Fig Uncropped images of the western blot gel. The expression levels of β-catenin, c-myc and GSK-3β were analyzed by western blot in AGS and BGC-823 cells. The cells were treated by Umbelliprenin (0, 6, 12 and 24 μM for AGS cells or 0, 12.5, 25 and 50 μM for BGC-823 cells). The tests were conducted in triplicate. A. The expression of β-catenin (cytosol) and c-myc in AGS cells after Umbelliprenin treatment (0, 6, 12, 24 μM) were detected by western blot. B. The expression of GSK-3β in BGC-823 cells after Umbelliprenin (0, 12.5, 25, 50 μM) treatment were detected by western blot.
(TIF)
